# Alternative Splicing of SCL30a Generates Distinct Isoforms to Modulate ABA Signaling in Arabidopsis

**DOI:** 10.3390/plants15111735

**Published:** 2026-06-03

**Authors:** Tiantian Wu, Ping Lin, Ying Li, Yuan Tian, Mohammad Saidur Rhaman, Fuyuan Zhu, Yinggao Liu, Yanjie Xie

**Affiliations:** 1 Laboratory Center of Life Sciences, College of Life Sciences, Nanjing Agricultural University, Nanjing 210095, China; 2 College of Life Science, Shandong Agricultural University, Tai’an 271018, China; 3 National Key Laboratory for the Development and Utilization of Forest Food Resources, Co-Innovation Center for Sustainable Forestry in Southern China, State Key Laboratory of Tree Genetics and Breeding, Key Laboratory of State Forestry and Grassland Administration on Subtropical Forest Biodiversity Conservation, College of Life Sciences, Nanjing Forestry University, Nanjing 210037, China; 4 Department of Seed Science and Technology, Bangladesh Agricultural University, Mymensingh 2202, Bangladesh; saidursst@bau.edu.bd

**Keywords:** SR protein, alternative splicing, NMD, ABA

## Abstract

Alternative splicing (AS) coupled with nonsense-mediated decay (NMD) is an important post-transcriptional mechanism that regulates the expression of many genes, including serine/arginine-rich (SR) proteins across eukaryotes. In plants, SR proteins participate in diverse developmental processes and stress responses, particularly in abscisic acid (ABA) signaling. However, the functional differences among individual splice isoforms of SR proteins remain poorly understood. Here, we investigated SCL30a, a plant-specific SR protein in *Arabidopsis thaliana*. By integrating third-generation long-read transcriptome sequencing, NMD stability assays, and subcellular localization analyses, we identified five alternatively spliced *SCL30a* transcripts. Among them, SCL30a.2 and SCL30a.3 contain premature termination codons (PTCs), display nucleocytoplasmic localization, and are rapidly degraded through the NMD pathway. In contrast, the other three isoforms, SCL30a.1, SCL30a.4, and SCL30a.5, retain an intact RS domain and localize exclusively to the nucleus. Functional analyses showed that SCL30a acts as a positive regulator of ABA signaling. Loss-of-function mutants of *SCL30a* displayed reduced ABA sensitivity in both root growth and seed germination assays, whereas complementation or overexpression of three stable isoforms of *SCL30a* (*SCL30a.1*, *SCL30a.4*, and *SCL30a.5*) enhanced ABA responsiveness. Transcriptome analysis further showed that the expression of a subset of ABA-related genes, particularly *SnRK2.6*, was significantly altered in ABA-treated *scl30a* mutants and *SCL30a.1*-OE lines compared with WT plants. In addition, genetic evidence showed that overexpression of *SnRK2.6* rescued the ABA-insensitive phenotype of the *scl30a* mutant. Together, these findings suggest that SnRK2.6 may function as a candidate downstream component associated with SCL30a-mediated ABA responses.

## 1. Introduction

AS is a fundamental mechanism that generates multiple transcripts from a single pre-mRNA, thereby expanding the coding capacity of the genome and enabling organisms to respond to complex environmental and developmental cues with a limited number of genes [1,2]. Among the important regulators of AS are SR proteins, a highly conserved family of splicing factors that recognize splice sites, recruit spliceosomal components, and regulate splice site selection [3,4]. Structurally, SR proteins contain one or two N-terminal RNA recognition motifs (RRMs) for binding splicing enhancers and a C-terminal RS (arginine/serine-rich) domain, which mediates protein–protein interactions and spliceosome assembly through dynamic phosphorylation and dephosphorylation [5,6]. Through their RS domains, SR proteins interact with U1-70K of U1 snRNP (small nuclear ribonucleoprotein U1) and U2AF of U2 snRNP (small nuclear ribonucleoprotein U2), forming a bridge between 5′ and 3′ splice sites to assemble the spliceosome on U2-type introns [7,8]. Beyond their canonical roles in splicing, SR proteins participate in multiple layers of gene expression regulation, including transcriptional elongation, mRNA nuclear export, translation, and RNA decay. For example, SRSF1 facilitates mRNA export and contributes to the NMD pathway of transcripts containing PTCs [9,10]. In addition, SR proteins interact with N6-methyladenosine (m6A) regulators, linking them to epitranscriptomic control [6]. Collectively, these diverse functions position SR proteins as central hubs that integrate transcription, RNA processing, and RNA quality control. Interestingly, the SR protein family comprises numerous members, among which the SCL subfamily is plant-specific [11]. SR-like proteins, such as SR45 and SR45a, play important roles in plant growth, development, and stress responses, and their expression and splicing activities are finely tuned by developmental and environmental cues [12,13]. These conserved yet adaptable features underscore the importance of plant-specific SR proteins, particularly members of the SCL subfamily, in coordinating gene regulatory networks.

A defining feature of SR genes is their coupling with the NMD pathway through AS, forming an autoregulatory AS-NMD circuit. SR genes frequently generate unproductive transcripts containing PTCs via AS, which are subsequently eliminated by NMD to maintain SR protein homeostasis [14,15,16]. Notably, all conserved SR proteins produce such NMD-targeted isoforms, often associated with ultraconserved elements [17,18]. This unproductive splicing represents an evolutionarily ancient yet flexible regulatory strategy, conserved from fungi to animals, that enables rapid turnover of transcripts and independent evolution of poison exons following gene duplication [19]. Temperature-controlled AS-NMD coupling is conserved in plants, acting as a clock-independent mechanism for rhythmic gene expression, while SR proteins form highly interconnected regulatory networks via AS-NMD [20,21,22].

In Arabidopsis, approximately 50% of alternatively spliced SR transcripts are predicted to be NMD targets, with several experimentally validated as bona fide substrates [19,23], and similar mechanisms control splicing factor expression in yeast [24]. Thus, AS-NMD coupling represents a central mechanism by which plant SR proteins achieve precise autoregulation. Importantly, SR proteins not only serve as substrates of NMD but also actively regulate this pathway. For instance, in mammals, SR proteins target PTC-containing mRNAs to NMD via their RS domain, and their overexpression enhances NMD efficiency, indicating that SR proteins serve both as NMD substrates and active NMD regulators, forming complex feedback networks [6]. In parallel, the subcellular localization of SR proteins is tightly linked to their function. Nuclear import depends on Transportin-SR family members that recognize phosphorylated RS repeats and mediate translocation through the nuclear pore [25]. Recent structural studies reveal that phosphorylation of the RS domain is essential for high-affinity binding to TNPO3, whereas unphosphorylated RS domains fail to bind effectively [26]. Moreover, RS domain phosphorylation is required for SR proteins to engage in RNA binding [7]. Consequently, deletion or altered phosphorylation of the RS domain leads to cytoplasmic retention and impaired splicing activity. This regulatory mechanism is conserved in plants; for example, the RS domains of splicing factors such as RS2Z32 and RS2Z33 are critical determinants of nuclear localization [27]. Taken together, the precise expression and function of SR proteins are coordinated by an evolutionarily conserved yet highly dynamic AS-NMD autoregulatory system, coupled with RS domain phosphorylation-dependent control of nuclear localization.

ABA is a central phytohormone that orchestrates plant stress responses, seed dormancy and germination, seedling growth, and stomatal movement [28]. The core ABA signaling module consists of ABA receptors (PYR/PYLs), clade A protein phosphatase 2Cs (PP2Cs), and downstream SnRK2 protein kinases (SnRK2s) [29]. Under basal conditions, PP2Cs directly interact with and inhibit SnRK2 activity through dephosphorylation [30]. Upon ABA perception, ABA-bound PYR/PYL receptors sequester PP2Cs, thereby releasing SnRK2s to phosphorylate downstream transcription factors such as ABI5 and activate ABA-responsive gene expression [31,32]. Accumulating evidence indicates that SR proteins play important regulatory roles in ABA signaling. For example, the Arabidopsis SR45 acts as a negative regulator, and ABA promotes its accumulation by inducing dephosphorylation, which inhibits ubiquitination and proteasomal degradation [33]. Several additional SR family members, including SR34, SR34b, SCL30a, RS40, and SR45a, have been implicated in ABA-mediated stress responses [34]. Notably, the splicing factor At-RS31 undergoes AS to generate four isoforms, among which the productive isoform coordinates TOR (Target of Rapamycin) and ABA signaling pathways by modulating the splicing of key ABA components such as *HAB1* and *SnRK2.8*, thereby fine-tuning the balance between growth and stress responses [35]. Similarly, ABA signaling in darkness promotes cotyledon closure via modulation of alternative splicing by RS40 and RS41, whereas light exposure reduces ABA levels, relieving this repression. In this process, the function of RS40 and RS41 is controlled by both transcriptional regulation and phosphorylation-dependent post-translational modifications [36]. Collectively, these studies establish SR proteins as key regulators of ABA signaling, acting through multiple molecular mechanisms at both the protein and transcript levels. Importantly, SR proteins also regulate the alternative splicing of ABA-responsive genes, thereby fine-tuning the amplitude and dynamics of ABA responses at the post-transcriptional level. This highlights a critical regulatory layer linking RNA processing to hormone signaling.

In this study, we focused on SCL30a, a member of the plant-specific SCL subfamily of SR proteins in *Arabidopsis thaliana*, to investigate whether and how distinct alternatively spliced isoforms of *SCL30a*, produced via AS-NMD, differentially regulate ABA signaling. By integrating third-generation transcriptome sequencing, NMD stability assays, and subcellular localization analysis, we demonstrate that *SCL30a* generates multiple transcripts through AS. Among these isoforms, those containing PTCs are selectively degraded via the NMD pathway, whereas the stable isoforms encode proteins that retain an intact RS domain and localize to the nucleus. Functional analyses further reveal that SCL30a acts as a positive regulator of ABA signaling and mediates ABA responses by modulating the expression of the downstream target gene *SnRK2.6*.

## 2. Results

### 2.1. SCL30a Generates Multiple Transcripts Through Alternative Splicing

To characterize the AS landscape in response to ABA, we previously conducted transcriptomic and proteomic analyses using 12-day-old Arabidopsis seedlings treated with ABA for 6 h and 48 h, together with untreated controls [37]. These analyses showed that approximately 78% of intron-containing genes undergo AS, underscoring the widespread involvement of AS in ABA responses. Among the ABA-responsive AS events identified, SCL30a attracted particular attention because it encodes a member of the plant-specific SCL subfamily of SR proteins.

**Figure 1:**
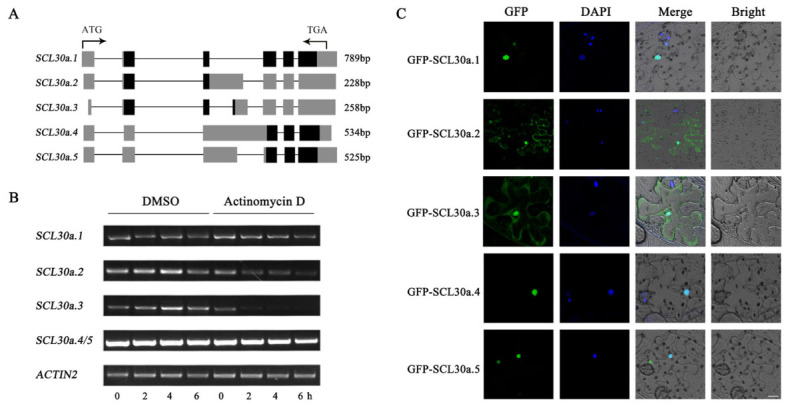
Expression profiles of different *SCL30a* transcripts. (**A**) Schematic representation of the *SCL30a* gene structure. *SCL30a* consists of exons (boxes) and introns (lines). Gray and black boxes represent untranslated regions (UTRs) and coding sequences (CDS), respectively. Alternative splicing generates five major transcripts (*SCL30a.1*–*5*), and their differential exon composition is shown. The number on the right of each transcript indicates the length (bp) of its coding region. (**B**) Nonsense-mediated mRNA decay (NMD) analysis. Arabidopsis seedlings were treated with DMSO (control) or actinomycin D, and the abundance of different *SCL30a* transcripts was examined at 0, 2, 4, and 6 h after treatment. *ACTIN2* served as a loading control. The PCR cycle numbers for the transcripts and the internal control were 32 and 26, respectively. (**C**) Subcellular localization of different transcripts in tobacco leaves. Different SCL30a transcripts were transiently expressed in tobacco (*Nicotiana benthamiana*) leaves. Nuclei were stained with DAPI. Scale bar = 50 μm.

To further resolve the transcript complexity of *SCL30a*, we performed third-generation long-read transcriptome sequencing and identified five distinct transcript isoforms (Figure 1A). Sequence analysis showed that two of these isoforms, *SCL30a.2* and *SCL30a.3*, contain PTCs within their coding regions, which would be predicted to produce truncated proteins if translated. PTC-containing transcripts are frequently recognized and degraded by the NMD pathway, a conserved RNA surveillance mechanism that prevents the accumulation of potentially deleterious truncated proteins [38,39,40].

To determine whether the different *SCL30a* isoforms differ in transcript stability and therefore in their potential function relevance, we performed transcriptional inhibition assays using actinomycin D in wild-type (WT) seedlings. By blocking *de novo* transcription, we monitored the decay kinetics of individual isoforms over time using semi-quantitative RT-PCR. Transcript abundance was examined at multiple time points after treatment, with untreated seedlings serving as controls. Under transcriptional arrest, *SCL30a.2* and *SCL30a.3* exhibited rapid and pronounced decay, indicating that these transcripts are intrinsically unstable. In contrast, isoforms *SCL30a.1*, *SCL30a.4*, and *SCL30a.5* remained relatively stable throughout the time course and showed no obvious reduction in abundance (Figure 1B). Taken together, these results revealed a clear functional divergence among *SCL30a* splice variants. The PTC-containing isoforms (*SCL30a.2* and *SCL30a.3*) are likely subjected to NMD and therefore unlikely to support stable protein production, whereas the PTC-free isoforms persist and may encode functional proteins.

### 2.2. The RS Domain Determines the Nuclear Localization of Stable SCL30a Isoforms

To further investigate the functional differences among the *SCL30a* splice isoforms, we examined their subcellular localization. GFP fusion constructs corresponding to each isoform were transiently expressed in *Nicotiana benthamiana* leaves through *Agrobacterium*-mediated infiltration. Confocal microscopy revealed that SCL30a.1, SCL30a.4, and SCL30a.5 localized predominantly to the nucleus, whereas SCL30a.2 and SCL30a.3 were detected in both the nucleus and the cytoplasm (Figure 1C). Notably, this localization pattern closely correlated with the stability profiles of the corresponding transcripts. The nucleocytoplasmic proteins (SCL30a.2/.3) are derived from unstable, NMD-sensitive transcripts, whereas nuclear-localized proteins (SCL30a.1/.4/.5) originate from stable transcripts. These findings indicate that alternatively spliced isoforms of *SCL30a* exhibit distinct subcellular localization patterns, suggesting potential functional diversification among the isoforms.

Previous studies have shown that the RS domain of SR proteins is involved not only in spliceosome assembly but also in nuclear localization [41,42]. To determine whether the distinct localization patterns of the SCL30a isoforms are associated with structural differences, we performed secondary structure prediction analysis of the five isoforms. The results showed that the nuclear-localized proteins (SCL30a.1/.4/.5) retain an intact RS domain, whereas the nucleocytoplasmic-localized proteins (SCL30a.2/.3) completely lack this domain (Figure S1A). This structural distinction is highly consistent with their respective localization patterns, suggesting that the RS domain is important for nuclear targeting of SCL30a proteins. Notably, the isoforms lacking the RS domain are also intrinsically unstable, consistent with our earlier finding that SCL30a.2 and SCL30a.3 are subjected to NMD-mediated degradation. To further validate these observations in a stable transgenic system, we generated Arabidopsis lines constitutively expressing GFP-tagged SCL30a.1, SCL30a.4, or SCL30a.5. Subcellular localization analysis in these stable transgenic lines was highly consistent with the transient expression results, with all three proteins exclusively localized to the nucleus (Figure S1B). These results further support the conclusion that the stable SCL30a isoforms maintain nuclear localization in planta.

We next investigated the spatial expression patterns of *SCL30a* isoforms across different Arabidopsis tissues. RT-PCR analyses showed that *SCL30a* transcripts are ubiquitously expressed in all tissues examined, including cauline leaves, rosettes, siliques, stems, shoots, roots, flowers, and seeds, with no obvious tissue-specific enrichment (Figure S2A). Consistent with these results, GUS staining analysis using *SCL30a* promoter-driven reporter lines revealed broad expression throughout seedlings, including cotyledons, hypocotyls, and roots (Figure S2B). This widespread expression pattern of SCL30a suggests that it may participate in fundamental physiological and developmental processes across diverse tissues and developmental stages.

### 2.3. SCL30a Is Involved in the ABA Response

Previous studies have suggested that SCL30a may participate in ABA response [34]. To further investigate this possibility, we examined the expression of *SCL30a* in Arabidopsis seedlings following ABA treatment. The results showed that ABA treatment significantly reduced *SCL30a* transcript abundance (Figure S3A), supporting a potential role for *SCL30a* in ABA responses. This ABA-induced downregulation also suggests that SCL30a expression may be negatively regulated by ABA response at either the transcriptional or post-transcriptional level. To further characterize the biological function of SCL30a, we obtained and characterized a T-DNA insertion mutant from the Arabidopsis Biological Resource Center (ABRC, Columbus, OH, USA), which was designated *scl30a* (Figure S3B). The insertion site was verified by PCR analysis, and RT-PCR using gene-specific primers spanning the coding region confirmed that *scl30a* is a knockout mutant, as no full-length transcript was detected (Figure S3C).

**Figure 2:**
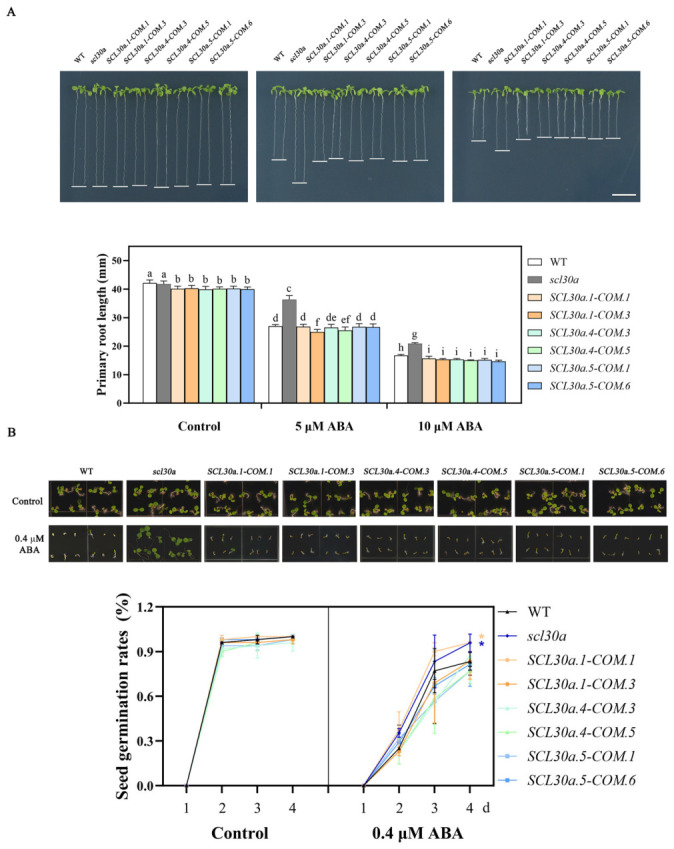
Root length and germination phenotypes of *SCL30a* mutant and genetic complementation lines under ABA treatment. (**A**) Root length phenotype. Representative images (**top**) and quantification (**bottom**) of root length in WT, *scl30a*, and genetic complementation lines under 0, 5, and 10 μM ABA. Data are shown as mean ± SE. Different lowercase letters indicate significant differences (*p* < 0.05). Scale bar = 1 cm. Data are from three independent biological replicates, with at least 10 plants scored per replicate. (**B**) Germination phenotype. Representative images (**top**) and germination rate quantification (**bottom**) of WT, *scl30a*, and genetic complementation lines under 0 μM and 0.4 μM ABA. Asterisks indicate significant differences compared with WT under the same ABA concentration (* *p* < 0.05). Data are from three independent biological replicates, with at least 50 seeds scored per replicate.

To systematically investigate the role of *SCL30a* in ABA response, we introduced the coding sequences of *SCL30a.1*, *SCL30a.4*, or *SCL30a.5* into the *scl30a* background. In parallel, *SCL30a*-COM lines for each isoform were generated under the control of the constitutive 35S promoter, and transgene expression was confirmed by RT-PCR (Figure S3C). These transgenic materials enabled us to assess both the necessity and sufficiency of individual *SCL30a* isoforms in ABA responses. We first evaluated their response to ABA-mediated inhibition of root elongation. Seedlings were grown vertically on medium containing different concentrations of ABA, and primary root length was measured after 5 days. Under ABA treatment, WT seedlings showed strong inhibition of root growth, whereas the *scl30a* mutant displayed significantly reduced sensitivity, as reflected by less pronounced inhibition of root elongation (Figure 2A). This ABA-insensitive phenotype suggests that loss of SCL30a impairs the ABA response during root growth. Importantly, expression of *SCL30a.1*, *SCL30a.4*, or *SCL30a.5* in the *scl30a* background restored ABA sensitivity compared to *scl30a* mutant, indicating that all three stable isoforms are functionally competent in mediating ABA-dependent root growth inhibition (Figure 2A). Together, these results demonstrate that the reintroduction of any of the three stable SCL30a isoforms effectively rescues the ABA-insensitive root elongation phenotype of the *scl30a* mutant.

We next investigated the ABA response during seed germination, another major developmental process regulated by ABA. Seeds from WT, *scl30a* mutant, and *SCL30a*-COM lines were sown on media with or without ABA, and germination rates were recorded daily. Consistent with the root elongation phenotype, ABA strongly inhibited germination in WT seeds, whereas *scl30a* mutants displayed significantly higher germination rates, indicating reduced sensitivity to ABA (Figure 2B). These results suggest that loss of *SCL30a* weakens the inhibitory effect of ABA on seed germination. Importantly, *SCL30a*-COM restored ABA sensitivity, effectively rescuing the ABA-insensitive germination phenotype of the mutant (Figure 2B). Together, these findings demonstrate all three stable *SCL30a* isoforms are capable of restoring normal ABA responsiveness during seed germination.

**Figure 3:**
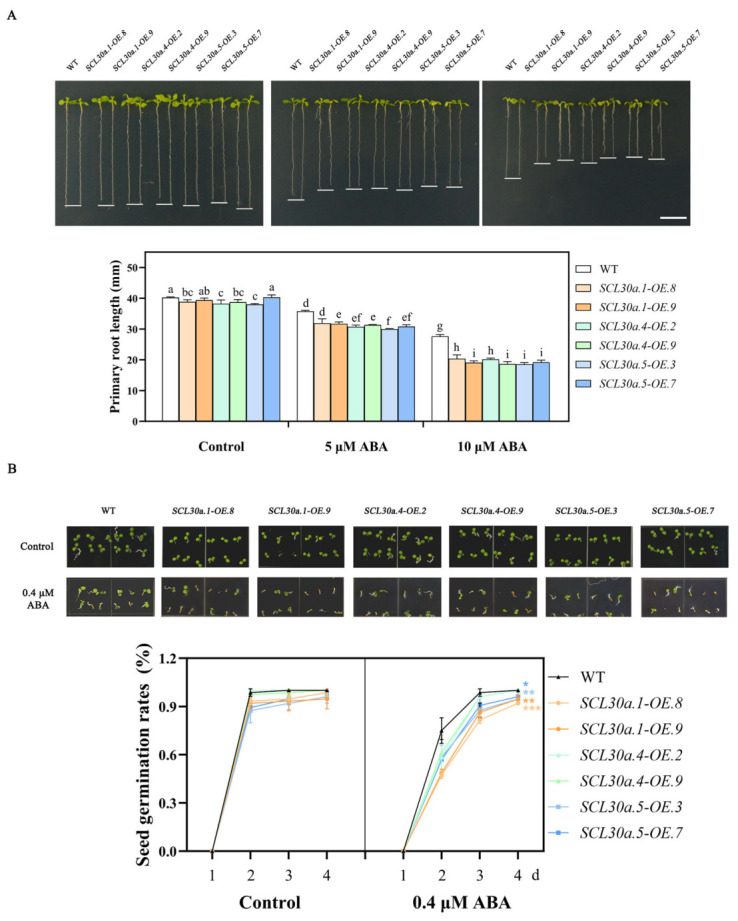
Root length and germination phenotypes of *SCL30a* overexpression lines under ABA treatment. (**A**) Root length phenotype. Representative images (**top**) and quantification (**bottom**) of root length in WT and *SCL30a* overexpression lines under 0, 5, and 10 μM ABA. Data are shown as mean ± SE. Different lowercase letters indicate significant differences (*p* < 0.05). Scale bar = 1 cm. Data are from three independent biological replicates, with at least 10 plants scored per replicate. (**B**) Germination phenotype. Representative images (**top**) and germination rate quantification (**bottom**) of WT and SCL30a overexpression lines under 0 μM and 0.4 μM ABA. Asterisks indicate significant differences compared with WT under the same ABA concentration (* *p* < 0.05, ** *p* < 0.01, *** *p* < 0.001). Data are from three independent biological replicates, with at least 50 seeds scored per replicate.

To determine whether increased *SCL30a* expression enhances ABA responses, we analyzed overexpression (*SCL30a*-OE) lines under the same experimental conditions. Under exogenous ABA treatment, *SCL30a*-OE seedlings exhibited significantly stronger inhibition of primary root elongation compared with WT seedlings, indicating enhanced sensitivity to ABA (Figure 3A). This result suggests that increased *SCL30a* expression potentiates ABA-mediated inhibition of root growth, consistent with a positive regulatory role in ABA response. Consistent with the root elongation phenotypes, seed germination assays showed that, on medium containing 0.4 μM ABA, *SCL30a*-OE lines displayed significantly lower germination rates than WT seeds (Figure 3B). These results further support the conclusion that *SCL30a* expression enhances ABA sensitivity during seed germination.

### 2.4. SCL30a Regulates Gene Expression Involved in ABA Response

To further investigate the molecular basis of SCL30a function in ABA response, we performed transcriptome sequencing using 10-day-old WT, *scl30a* mutant, and *SCL30a.1*-OE seedlings treated with 50 μM ABA for 4 h. Principal component analysis (PCA) showed that biological replicates from each genotype clustered closely together, indicating high reproducibility and reliability of the transcriptomic data. In addition, the three genotypes were clearly separated along the principal components under ABA treatment (Figure S4A), suggesting that WT, *scl30a* mutant, and *SCL30a.1*-OE lines exhibit distinct transcriptomic profiles in response to ABA. Differential expression analysis identified 5518 differentially expressed genes (DEGs) in the *scl30a* mutant and 7674 DEGs in the *SCL30a.1*-OE line compared with WT. Among these, 155 genes in the *scl30a* mutant and 189 genes in the *SCL30a.1*-OE line were putatively associated with ABA response (Figure S4B).

**Figure 4:**
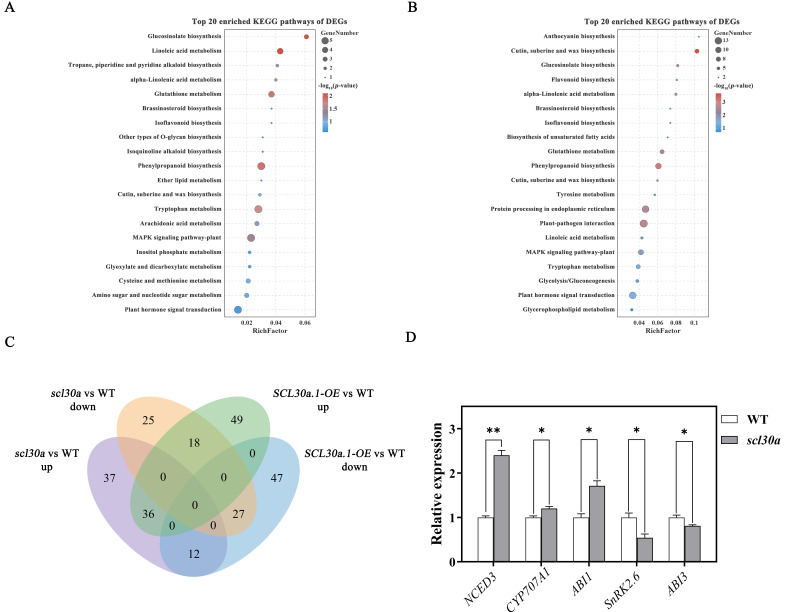
Transcriptome analysis of *SCL30a* mutant and overexpression lines under ABA treatment. (**A**) KEGG enrichment of DEGs in the mutant. KEGG pathway enrichment analysis of DEGs in *scl30a* compared with WT under ABA treatment. (**B**) KEGG enrichment of DEGs in the overexpression line. KEGG pathway enrichment analysis of DEGs in the *SCL30a* overexpression line (*SCL30a.1-OE*) compared with WT under ABA treatment. (**C**) Venn diagram of DEGs. Overlap of DEGs identified in *scl30a* and *SCL30a.1-OE*, each compared with WT, under ABA treatment. The numbers of unique and shared DEGs in each set are indicated. (**D**) qRT-PCR validation of ABA-related genes. Expression levels of genes involved in ABA biosynthesis (*NCED3*), metabolism (*CYP707A1*), and signaling (*ABI1*, *SnRK2.6*, *ABI3*) in *scl30a* compared with WT. Data are shown as mean ± SE from at least three independent biological replicates. *ACTIN2* and *GAPDH* were used as reference genes. Asterisks indicate significant differences compared with WT (* *p* < 0.05, ** *p* < 0.01).

Functional enrichment analysis showed that the DEGs identified in the *scl30a* mutant were mainly enriched in pathways related to Glucosinolate biosynthesis, Linoleic acid metabolism, Tropane, piperidine and pyridine alkaloid biosynthesis, alpha-linolenic acid metabolism, Glutathione metabolism, Brassinosteroid biosynthesis, and plant hormone signal transduction (Figure 4A). In the *SCL30a.1*-OE line, the DEGs were predominantly enriched in pathways associated with Anthocyanin biosynthesis, Cutin, suberine and wax biosynthesis, Glutathione metabolism, Flavonoid biosynthesis, alpha-linolenic acid metabolism, Brassinosteroid biosynthesis, and plant hormone signal transduction (Figure 4B). To identify genes potentially associated with SCL30a-mediated regulation, we further compared the transcriptomic datasets from the loss- and gain-of-function lines. This analysis identified 18 genes that were downregulated in the *scl30a* mutant but upregulated in the *SCL30a.1*-OE line, as well as 12 genes showing the opposite expression pattern (upregulated in the mutant and downregulated in the overexpression line) (Figure 4C, Table S1). The opposite expression trends observed between the *scl30a* mutants and *SCL30a.1*-OE line, suggesting that these genes may represent downstream components associated with SCL30a-dependent regulatory processes. By comparing these candidates with previously characterized ABA response genes, we identified *NCED3*, *CYP707A1*, *ABI3*, *ABI1*, and *SnRK2.6* as candidate downstream target genes potentially associated with *SCL30a* (Figure 4D). Among them, SnRK2.6, also known as OPEN STOMATA 1 (OST1), was significantly downregulated in ABA-treated *scl30a* mutants compared with WT (Figure 4D). Given that SnRK2.6 is a central positive regulator of ABA signaling, its reduced expression is consistent with the ABA-insensitive phenotypes observed in the *scl30a* mutant during root elongation and seed germination.

### 2.5. SnRK2.6 Participates in the ABA Responses Regulated by SCL30a

**Figure 5:**
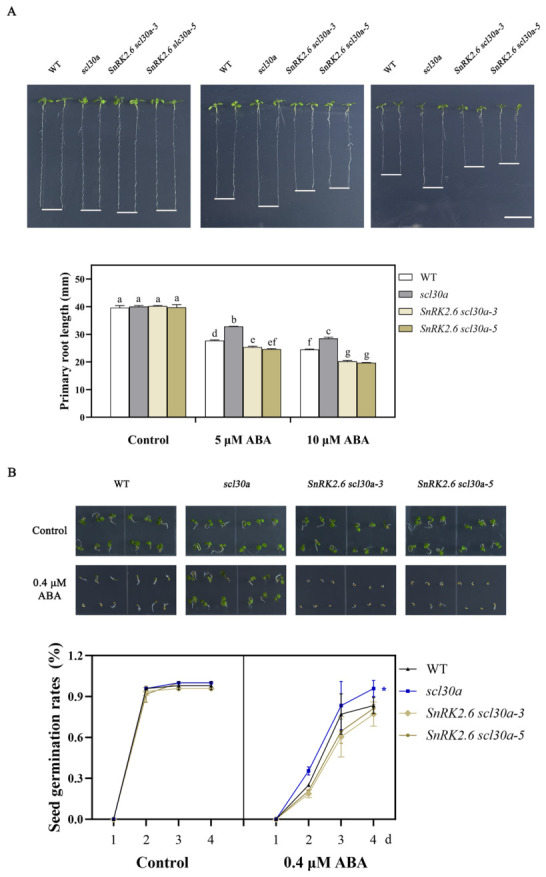
Root length and germination phenotypes of *SnRK2.6* complementation in the *scl30a* mutant under ABA treatment. (**A**) Root length phenotype. Representative images (**top**) and quantification (**bottom**) of root length in WT, *scl30a*, and *SnRK2.6* complementation transgenic lines (*SnRK2.6 scl30a*) under 0, 5, and 10 μM ABA. Data are shown as mean ± SE. Different lowercase letters indicate significant differences (*p* < 0.05). Scale bar = 1 cm. Data are from three independent biological replicates, with at least 10 plants scored per replicate. (**B**) Germination phenotype. Representative images (**top**) and germination rate quantification (**bottom**) of WT, *scl30a*, and *SnRK2.6/scl30a* complementation lines under 0 μM and 0.4 μM ABA. Asterisks indicate significant differences compared with WT under the same ABA concentration (* *p* < 0.05). Data are from three independent biological replicates, with at least 50 seeds scored per replicate.

To investigate the genetic relationship between SnRK2.6 and SCL30a in ABA signaling, we overexpressed *SnRK2.6* in the *scl30a* mutant background. The coding sequence of *SnRK2.6* was placed under the control of the constitutive 35S promoter and introduced into the *scl30a* mutant. Transgenic lines were selected and validated by RT-PCR and qRT-PCR analyses, which confirmed that *SnRK2.6* expression was restored in the *SnRK2.6* *scl30a* lines compared with the *scl30a* mutant under 10 μM ABA treatment (Figure S5). This restoration effectively compensated for the reduced *SnRK2.6* expression observed in the *scl30a* mutant. SnRK2.6 is a central protein kinase in the ABA signaling pathway that activates downstream transcription factors and regulates physiological processes such as stomatal closure, stress responses, and seed germination upon ABA perception [43]. Therefore, the reduced expression of *SnRK2.6* in the *scl30a* mutant may contribute to its ABA-insensitive phenotype. To test this possibility, we performed phenotypic analyses of the *SnRK2.6* complemented lines under ABA treatment, focusing on comparisons between the *SnRK2.6 scl30a* lines and the *scl30a* mutant. In root elongation assays, the *scl30a* mutant displayed reduced sensitivity to ABA, as reflected by weaker inhibition of root growth compared to WT seedlings. In contrast, the *SnRK2.6 scl30a* lines exhibited significantly stronger inhibition of primary root elongation than the *scl30a* mutant (Figure 5A), indicating restoration of ABA sensitivity. Consistent with this result, seed germination assays showed that the *scl30a* mutant maintained a high germination rate under ABA treatment, whereas the *SnRK2.6 scl30a* lines displayed significantly reduced germination rate compared with the *scl30a* mutant (Figure 5B). Together, these results suggest that *SnRK2.6* contributes to SCL30a-mediated ABA responses and may function as a downstream component in this regulatory pathway.

## 3. Discussion

In this study, we combined third-generation transcriptome sequencing with NMD stability assays to show that *SCL30a* generates five distinct transcripts with differential fates. Among these, *SCL30a.2* and *SCL30a.3* contain PTCs and are degraded through the NMD pathway, whereas *SCL30a.1*, *SCL30a.4*, and *SCL30a.5* are relatively stable (Figure 1A,B). Consistent with these differences in transcript stability, subcellular localization analysis showed that proteins encoded by the stable transcripts predominantly localize to the nucleus, whereas those derived from unstable transcripts display a nucleocytoplasmic distribution (Figure 1C). Sequence analysis further revealed that the nuclear-localized isoforms retain an intact RS domain. Phenotypic analyses showed that the *scl30a* mutant exhibits reduced ABA sensitivity during both root elongation and seed germination, whereas expression of *SCL30a.1*, *SCL30a.4*, or *SCL30a.5* in the mutant background restored ABA responsiveness (Figure 2). In addition, *SCL30a*-OE lines displayed enhanced ABA sensitivity (Figure 3), supporting a positive role of SCL30a in ABA response. Transcriptome analysis further showed that SCL30a-responsive genes are enriched in pathways related to plant hormone signal transduction, spliceosome function, and diverse metabolic processes. Among the candidate genes identified, SnRK2.6, a core kinase in ABA signaling, showed altered expression in the *scl30a* mutants (Figure 4). Moreover, overexpression of *SnRK2.6* in the *scl30a* mutant restored ABA responsiveness (Figure 5), suggesting that SCL30a mediates ABA signaling through *SnRK2.6*. Together, these findings suggest that SCL30a generates alternatively spliced isoforms with distinct stability and subcellular localization associated with the presence or absence of the RS domain, and SCL30a participates in ABA responses, potentially through modulation of *SnRK2.6* expression.

The coupling of AS with NMD represents an important regulatory mechanism for gene expression in eukaryotes and plays a central role in the autoregulation of splicing factors [44]. In this study, using the Arabidopsis SR protein SCL30a as a model, we show that distinct alternatively spliced transcripts are differentially partitioned into the NMD pathway and that their encoded proteins exhibit distinct subcellular localization patterns associated with the presence or absence of the RS domain. These observations are consistent with previous studies of SR protein autoregulation in both plant and animal systems and further support the functional diversification of SR protein isoforms. We found that *SCL30a* generates five alternatively spliced isoforms, among which *SCL30a.2* and *SCL30a.3* contain PTCs and are likely targeted for degradation through NMD. This result is consistent with the study by Reddy and Palusa showing that half of Arabidopsis SR genes produce splice isoforms that are potential NMD substrates, including 25 experimentally validated targets [23]. Similarly, Zhang and Krainer demonstrated in the mammalian system that SR proteins can promote the degradation of PTC-containing transcripts through the NMD pathway, and that elevated SR protein levels enhance NMD efficiency [17]. Together, these findings suggest that the NMD sensitivity of *SCL30a.2* and *SCL30a.3* may represent a conserved autoregulatory mechanism contributing to the maintenance of *SCL30a* homeostasis. In addition, we observed clear differences in the subcellular localization of proteins encoded by distinct *SCL30a* isoforms, which correlate with both their NMD sensitivity and the integrity of the RS domain. Nuclear import of SR proteins is known to depend on recognition of phosphorylated RS domain by Transportin-SR family members, which mediate transport through the nuclear pore complex [17], and recent structural studies further support the role of TNPO3 in this process [26]. Therefore, the nucleocytoplasmic localization of SCL30a.2 and SCL30a.3, which lack the RS domain, is consistent with the established mechanism of SR protein nuclear import. These findings show a finely tuned regulatory model in which AS, NMD, and RS domain-mediated nuclear localization collectively influence the fate of *SCL30a* transcripts. In this model, isoforms containing PTCs and lacking the RS domain (SCL30a.2/.3) are preferentially degraded through NMD and display nucleocytoplasmic localization if translated, whereas isoforms retaining an intact RS domain (SCL30a.1/.4/.5) are relatively stable and predominantly nuclear localized. Nevertheless, whether the production of *SCL30a.2* and *SCL30a.3* contributes to the regulation of functional *SCL30a* isoforms remains unclear and requires further investigation.

Phenotypic analyses in this study support a positive role of SCL30a in ABA signaling across multiple developmental stages. The *scl30a* mutant displayed reduced ABA sensitivity during both root elongation and seed germination, whereas *SCL30a*-COM lines restored ABA responsiveness. In contrast, *SCL30a*-OE lines exhibited enhanced ABA sensitivity (Figure 2 and Figure 3). These observations suggest that SCL30a participates in ABA responses during both root growth and seed germination, suggesting that SCL30a may function at an important regulatory point in the ABA signaling pathway. Previous studies have shown that different SR proteins exert distinct effects on ABA signaling [34]. For example, overexpression of the cassava RSZ subfamily member *MeRSZ21b* in Arabidopsis enhances ABA sensitivity during seed germination and seedling growth and improves drought tolerance [45]. Similarly, the Arabidopsis SR1 (CAMTA3) positively regulates ABA signaling by alleviating salicylic acid-mediated antagonism, as *sr1* mutants exhibit ABA insensitivity and increased drought susceptibility [46]. In contrast, the SR protein SR45 negatively regulates ABA signaling. ABA promotes SR45 accumulation through dephosphorylation, thereby stabilizing SR45 and suppressing early seedling development, while *sr45-1* mutants are hypersensitive to ABA [33]. The *sr45-1* mutant is hypersensitive to ABA, whereas *SR45* overexpression reduces ABA sensitivity [47,48]. These contrasting observations indicate that different SR proteins likely regulate distinct components or processes within the ABA pathway, thereby contributing to the fine-tuning of ABA responses. As a member of the plant-specific SCL subfamily, SCL30a appears to function similarly to positive regulators such as MeRSZ21b and SR1, but differently from SR45. This functional diversity may reflect the complex regulations underlying plant development and stress adaptation. Consistent with this possibility, another SCL subfamily member, MeSCL33, binds to the pre-mRNA of *MeABA1*, regulates its AS and expression, and thereby influence endogenous ABA levels and postharvest physiological deterioration resistance in cassava [49]. In our study, *SnRK2.6* expression was significantly reduced in the *scl30a* mutant, and overexpression of *SnRK2.6* in the *scl30a* mutant background restored ABA sensitivity during both root elongation and seed germination (Figure 5). These results suggest that SCL30a may influence ABA responses through directly or indirectly modulating the splicing or expression of *SnRK2.6*. In addition, several other ABA-related genes were identified, including *NCED3*, *CYP707A1*, *ABI3*, and *ABI1*, also displayed altered expression patterns consistent with the observed ABA phenotypes, suggesting that SCL30a may affect ABA signaling through multiple downstream effectors. Notably, ABA treatment reduced *SCL30a* transcript abundance, implying the existence of a feedback regulatory mechanism that may coordinate with the AS-NMD autoregulatory pathway. How ABA signaling regulates *SCL30a* expression and whether SCL30a directly modulates the splicing of ABA-related targets remain important questions for future investigation.

ABA is a central regulator of plant responses to diverse environmental stresses, including drought, salinity, and cold [50]. Because SCL30a positively influences ABA responses and affects the expression of *SnRK2.6*, it is possible that SCL30a also contributes to plant adaptation to multiple ABA-related stresses. SnRK2.6 functions not only as a core kinase in ABA signaling but also as an integrator of several stress-response pathways. Previous studies have shown that SnRK2.6 can be activated by osmotic stress, salinity, and cold, thereby promoting stomatal closure, stress-responsive gene expression, and regulation of seed germination [51,52,53,54]. Therefore, the altered expression of *SnRK2.6* observed in the *scl30a* mutant may contribute to broader changes in stress responsiveness. Interestingly, the rice homolog *OsSCL30* has been reported to negatively regulate tolerance to low temperature, drought, and salt [55]. One possible explanation for the broad regulatory potential of SCL30a is its role as a splicing factor, since alternative splicing provides a rapid and reversible mechanism for modulating gene expression in response to environmental changes. Consistent with this idea, transcriptome enrichment analysis in this study showed that SCL30a-responsive genes are associated with multiple metabolic and hormone signaling pathways. In addition, previous studies have highlighted extensive connections between SR proteins and stress responses. Duque and colleagues proposed that plant SR proteins are regulated at multiple levels by environmental signals and are increasingly linked to stress adaptation [56], while Cruz and colleagues identified SCL30a and several other SR proteins as candidates involved in ABA-mediated stress responses [34]. Notably, the regulatory effect of SCL30a may vary depending on developmental stage and environmental conditions. In our study, SCL30a acts as a positive regulator of ABA responses during seedling growth and seed germination. However, Laloum and colleagues reported that *scl30a* mutant seeds display hypersensitivity to ABA and salt, suggesting a negative regulatory role for SCL30a during seed germination [57]. This difference may reflect variation in experimental conditions or developmental contexts. Alternatively, SCL30a may fine-tune ABA sensitivity according to the intensity or duration of stress signals. Overall, our findings support a role for SCL30a in ABA-associated stress responses and provide a basis for future studies investigating how SCL30a interacts with other stress-responsive pathways and regulatory factors during plant adaptation to environmental stress.

## 4. Materials and Methods

### 4.1. Plant Materials and Growth Conditions

The *Arabidopsis thaliana* mutant *scl30a* (SALK_041849; Col-0 ecotype) was obtained from the Arabidopsis Biological Resource Center (ABRC; http://www.arabidopsis.org/abrc (accessed on 20 December 2025)).

Seeds were surface-sterilized, washed three times with sterile water, and sown on solid half-strength Murashige and Skoog (1/2 MS) medium (pH 5.8). Plants were grown in a growth chamber under a 16 h light/8 h dark photoperiod (23 °C/18 °C, day/night) with a light intensity of 100 µmol photons m^−2^ s^−1^.

For germination assays, seeds were stratified at 4 °C for 72 h and then sown on 1/2 MS medium supplemented with or without 0.4 µM ABA. Germination rates were determined after the indicated periods.

For root growth assays, five-day-old seedlings of each genotype were transferred to 1/2 MS medium containing the indicated concentrations of ABA or no ABA. After cultivation for the specified durations, primary root lengths were measured, and representative images were taken.

### 4.2. Plasmid Construction and Plant Transformation

The coding sequences of the different *SCL30a* transcript isoforms and of *SnRK2.6* were amplified by PCR from *Arabidopsis thaliana* WT cDNA, whereas the coding sequence of *SCL30a.5* was synthesized in vitro. The resulting amplicons were cloned into binary vectors such as pMDC32, pMDC43, and pMDC163. The validated recombinant plasmids were introduced into *Agrobacterium tumefaciens* strain GV3101. Arabidopsis plants were transformed using the floral dip method. Transgenic plants were selected on 1/2 MS medium supplemented with hygromycin B (50 mg/L). The presence and expression of the transgenes were confirmed by RT-PCR, qRT-PCR, and fluorescence imaging.

### 4.3. Actinomycin D Treatment for NMD Analysis

To investigate whether different transcript isoforms of *SCL30a* are subject to nonsense-mediated mRNA decay (NMD), we treated 10-day-old *Arabidopsis thaliana* seedlings grown on 1/2 MS medium with Actinomycin D to inhibit transcription. Seedlings were transferred to liquid 1/2 MS medium containing 100 μg/mL Actinomycin D and sampled at 0, 2, 4, and 6 h post-treatment. Seedlings incubated in liquid 1/2 MS medium supplemented with an equal volume of DMSO (the solvent of Actinomycin D) were sampled in parallel at the same time points and served as negative controls. Samples collected at each time point were immediately frozen in liquid nitrogen and stored at −80 °C until further use.

### 4.4. Subcellular Localization Analysis

To analyze subcellular localization, full-length coding sequences of different transcript isoforms of *SCL30a* were cloned into the pMDC43 vector to generate N-terminal GFP fusion constructs. *Agrobacterium tumefaciens* strain GV3101 harboring the recombinant plasmids was cultured, and bacterial cells were collected and resuspended in infiltration buffer containing 10 mM MES (pH 5.6), 10 mM MgCl_2_, and 150 μM acetosyringone to a final OD_600_ of 0.4. The suspensions were infiltrated into leaves of *Nicotiana benthamiana* using a needleless syringe. After infiltration, plants were incubated in darkness at 25 °C for 48 h to allow for transient expression.

For subcellular localization observation, infiltrated leaf sections (approximately 0.5 cm × 0.5 cm) were excised and mounted in PBS buffer. Fluorescence signals of the target protein-GFP fusions were visualized using a laser scanning confocal microscope (LSM900) with excitation/emission wavelengths of 488 nm/500−530 nm.

For nuclear counterstaining, leaf samples were incubated in PBS buffer containing 1 μg/mL DAPI (4′,6-diamidino-2-phenylindole) for 5−10 min at room temperature in the dark, followed by two washes with PBS. DAPI fluorescence was detected with excitation/emission wavelengths of 405 nm/420−480 nm. GFP and DAPI channel images were captured and merged to confirm nuclear localization.

For fluorescence imaging of stable transgenic Arabidopsis plants, 4-day-old seedlings were used, and GFP fluorescence signals in root tips were observed and captured. All imaging experiments were performed with at least three independent biological replicates, and representative images are shown.

### 4.5. GUS Staining

Arabidopsis plants carrying the *pSCL30a*::GUS reporter construct were grown on 1/2 MS medium plates at 22 °C. To examine the expression pattern of the *SCL30a* promoter, seedling tissues were subjected to histochemical GUS staining using a GUS staining kit (Solarbio, Beijing, China). After staining, tissues were destained with 70% ethanol to remove chlorophyll and photographed using a stereomicroscope (Stemi 2000-C, Carl Zeiss, Oberkochen, Germany).

### 4.6. RT-PCR Analysis

To investigate AS patterns, total RNA was extracted from three independent biological replicates and analyzed by RT-PCR. PCR products were separated by electrophoresis on 2.5% (*w*/*v*) agarose gels. *ACTIN2* was used as a reference gene to normalize cDNA loading amounts across samples. Gels were visualized under UV light, and images were captured using a gel documentation system. Representative gel images from three independent biological replicates are shown. Gene-specific primers used for RT-PCR are listed in Table S2.

### 4.7. qRT-PCR Analysis

Total RNA was extracted from *Arabidopsis thaliana* seedlings of different genotypes using Universal RNA Extraction Reagent (Vazyme, Nanjing, China) according to the manufacturer’s instructions. Following reverse transcription to synthesize cDNA, qRT-PCR was performed using the ChamQ Universal SYBR qPCR Master Mix (Vazyme, Nanjing, China) in a 20 μL reaction system on a QuantStudio 5 real-time PCR system (Thermo Fisher Scientific, Waltham, MA, USA). Gene-specific primers used for qRT-PCR are listed in Table S2. Relative expression levels of target genes were calculated using the 2^−ΔΔCt^ method, with *ACTIN2* and *GAPDH* as reference genes to normalize gene expression data. Three biological replicates were performed for each experiment, with each biological replicate consisting of three technical replicates.

### 4.8. Transcriptome Analysis

Total RNA was extracted from 10-day-old seedlings of WT, *scl30a* mutant, and *SCL30a.1*-OE lines treated with 50 μM ABA for 4 h. RNA integrity and purity were assessed by agarose gel electrophoresis and Nanodrop spectrophotometer. Paired-end (150 bp) sequencing was performed on the Illumina NovaSeq 6000 platform. Raw reads were subjected to quality control to remove adapter-containing reads and low-quality reads. Clean reads were aligned to the reference genome using HISAT2. Gene expression levels were quantified using featureCounts, and transcript abundance was normalized as transcripts per million (TPM). Three independent biological replicates were used for each genotype. Differentially expressed genes (DEGs) were identified using DESeq2 with a threshold of |log_2_(fold change)| > 1 and an adjusted *p*-value < 0.05.

### 4.9. Statistical Analysis

All experiments were performed with at least three independent biological replicates. Statistical analyses were conducted using SPSS 22.0 software (IBM Corp., Armonk, NY, USA). Data are presented as mean ± standard error (SE). Comparisons between two groups were performed using Student’s *t*-test, while comparisons among multiple groups were performed using one-way analysis of variance (ANOVA) followed by both Fisher’s least significant difference (LSD) and Duncan’s multiple range test (DMRT) as post hoc tests. A *p* < 0.05 was considered statistically significant, and *p* < 0.01 and < 0.001 were considered highly significant. Graphs were generated using GraphPad Prism 8.0 software (GraphPad Software, Boston, MA, USA).

## Data Availability

The original contributions presented in this study are included in the article/Supplementary Material. Further inquiries can be directed to the corresponding authors.

## Supplementary Materials

The following supporting information can be downloaded at: https://www.mdpi.com/article/10.3390/plants15111735/s1, Figure S1: Protein secondary structure prediction and subcellular localization of different *SCL30a* transcripts in transgenic plants (A) Protein secondary structure prediction. Predicted secondary structures of proteins encoded by different *SCL30a* transcripts (*SCL30a.1*–*SCL30a.5*). The RNA recognition motif (orange) and RS domain (yellow) is indicated for each transcript. (B) Subcellular localization in stable transgenic plants. Subcellular localization of SCL30a proteins in root tips of transgenic plants expressing transcripts SCL30a.1, SCL30a.4, and SCL30a.5. A transgenic line expressing free GFP alone is shown as a control. Scale bar = 100 μm; Figure S2: Tissue expression profiles of different *SCL30a* transcripts and GUS staining analysis (A) Tissue expression levels of different transcripts. RT-PCR analysis of different *SCL30a* transcripts in various tissues of WT. *ACTIN2* served as a loading control. (B) GUS staining analysis. GUS staining of various tissues in transgenic plants carrying the *pSCL30a*::GUS reporter construct. Scale bar = 1 mm; Figure S3: Expression analysis of different *SCL30a* transcripts under ABA treatment and transgenic identification (A) Expression levels of different transcripts under ABA treatment. qRT-PCR analysis of different *SCL30a* transcripts (*SCL30a.1*–*SCL30a.5*) in WT under control and ABA-treated conditions. Data are shown as mean ± SE from at least three independent biological replicates. *ACTIN2* and *GAPDH* were used as reference genes. Different letters indicate significant differences (*p* < 0.05) between control and ABA treatment for each transcript. (B) Schematic of the T-DNA insertion site. Diagram showing the T-DNA insertion position in *scl30a* (indicated by a red triangle). (C) Expression identification in different transgenic plants. RT-PCR analysis of transcripts *SCL30a.1*, *SCL30a.4*, and *SCL30a.5* in WT, *scl30a*, and genetic complementation lines. *ACTIN2* served as a loading control; Figure S4: Principal component analysis and Venn diagram of differentially expressed genes from transcriptome sequencing (A) Principal component analysis (PCA). PCA of transcriptome samples from WT, *scl30a*, and *SCL30a.1-OE* under ABA treatment. The clustering distribution of each sample is indicated. (B) Venn diagram of differentially expressed genes (DEGs). Overlap of three gene sets: DEGs from the *scl30a* mutant, DEGs from the *SCL30a.1* -OE line, and known ABA signaling pathway-related genes (curated from the literature). The numbers of unique and shared genes in each set are indicated; Figure S5: Molecular identification of *SnRK2.6* transgenic plants (A) qRT-PCR expression analysis. Expression levels of *SnRK2.6* in WT, *scl30a*, and *SnRK2.6* complementation lines (*SnRK2.6 scl30a*). Data are shown as mean ± SE from at least three independent biological replicates. *ACTIN2* and *GAPDH* were used as reference genes. Different letters indicate significant differences (*p* < 0.05) among genotypes. (B) RT-PCR identification. RT-PCR analysis of *SnRK2.6* transcripts in WT, *scl30a*, and *SnRK2.6/scl30a* complementation lines. *ACTIN2* served as a loading control; Table S1: Genes with opposite expression in the *scl30a* mutant and OE lines; Table S2: Primers used in the article.
