# Supplementary material for: Alternative Splicing of SCL30a Generates Distinct Isoforms to Modulate ABA Signaling in Arabidopsis
Source: Plants (Basel). 2026 Jun 3;15(11):1735. doi: 10.3390/plants15111735 (PMC13259279; doi:10.3390/plants15111735)
Supplement: Supplementary file 1 [file plants-15-01735-s001.zip › S Figures.pdf]

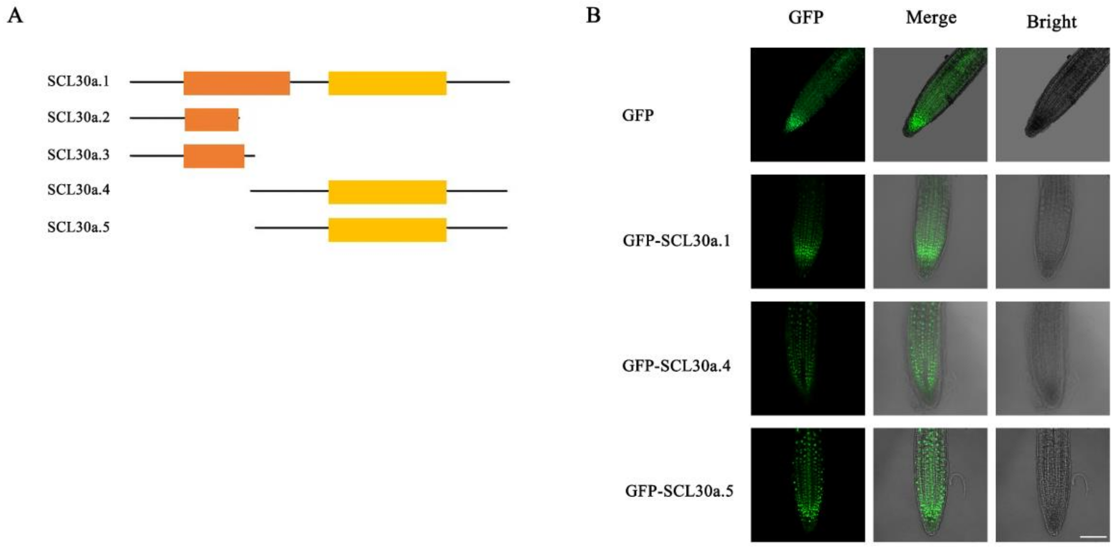


**Figure S1.** Protein secondary structure prediction and subcellular localization of different *SCL30a* transcripts in transgenic plants

**(A)** Protein secondary structure prediction. Predicted secondary structures of proteins encoded by different *SCL30a* transcripts (*SCL30a.1*-*SCL30a.5*). The RNA recognition motif (orange) and RS domain (yellow) is indicated for each transcript.

**(B)** Subcellular localization in stable transgenic plants. Subcellular localization of SCL30a proteins in root tips of transgenic plants expressing transcripts SCL30a.1, SCL30a.4, and SCL30a.5. A transgenic line expressing free GFP alone is shown as a control. Scale bar = 100 μm.

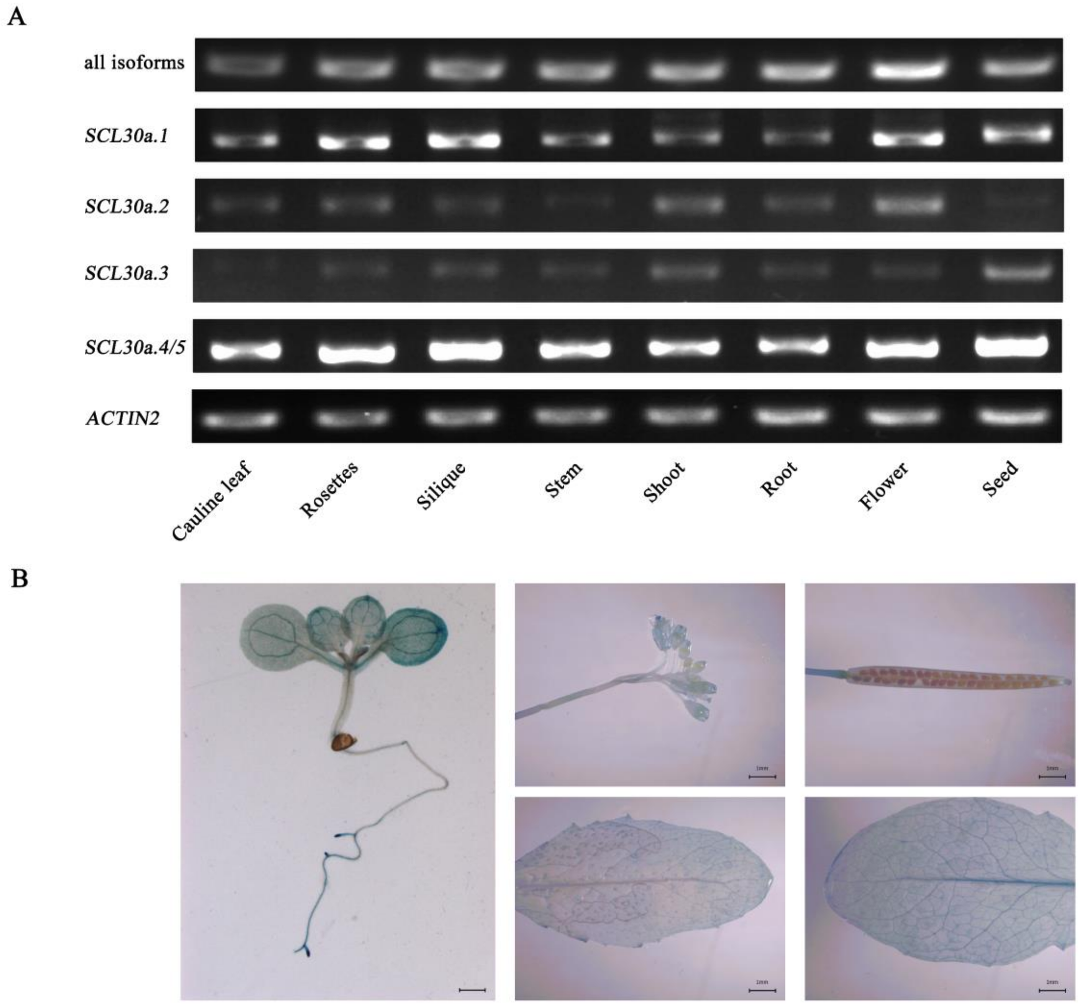


**Figure S2.** Tissue expression profiles of different *SCL30a* transcripts and GUS staining analysis

**(A)** Tissue expression levels of different transcripts. RT-PCR analysis of different *SCL30a* transcripts in various tissues of WT. *ACTIN2* served as a loading control.

**(B)** GUS staining analysis. GUS staining of various tissues in transgenic plants carrying the *pSCL30a*::GUS reporter construct. Scale bar = 1 mm.

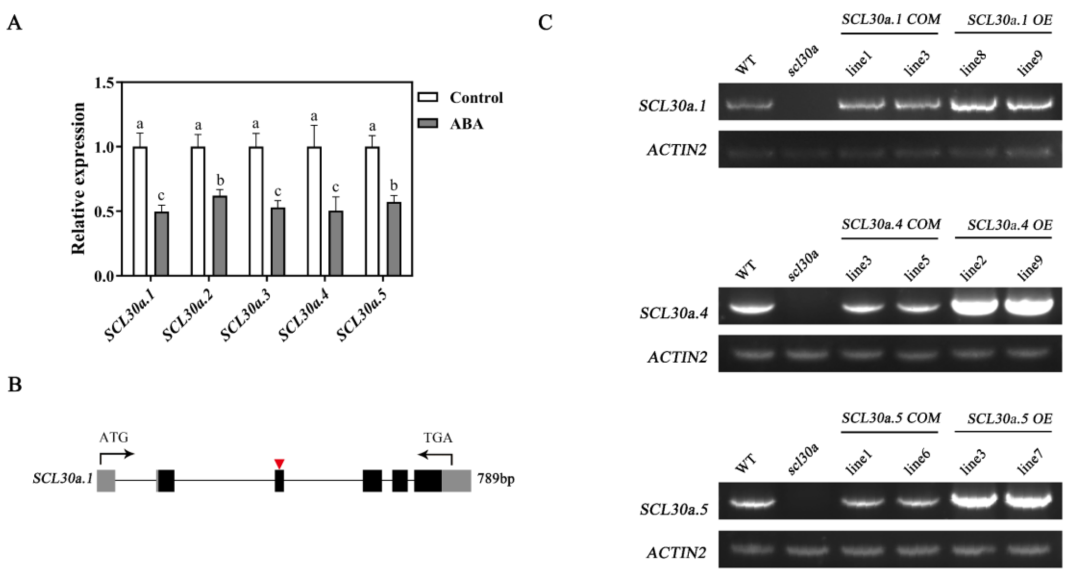


**Figure S3.** Expression analysis of different *SCL30a* transcripts under ABA treatment and transgenic identification

**(A)** Expression levels of different transcripts under ABA treatment. qRT-PCR analysis of different *SCL30a* transcripts (*SCL30a.1-SCL30a.5*) in WT under control and ABA-treated conditions. Data are shown as mean ± SE from at least three independent biological replicates. *ACTIN2* and *GAPDH* were used as reference genes. Different letters indicate significant differences ($p < 0.05$) between control and ABA treatment for each transcript.

**(B)** Schematic of the T-DNA insertion site. Diagram showing the T-DNA insertion position in *scl30a* (indicated by a red triangle).

**(C)** Expression identification in different transgenic plants. RT-PCR analysis of transcripts *SCL30a.1*, *SCL30a.4*, and *SCL30a.5* in WT, *scl30a*, and genetic complementation lines. *ACTIN2* served as a loading control.

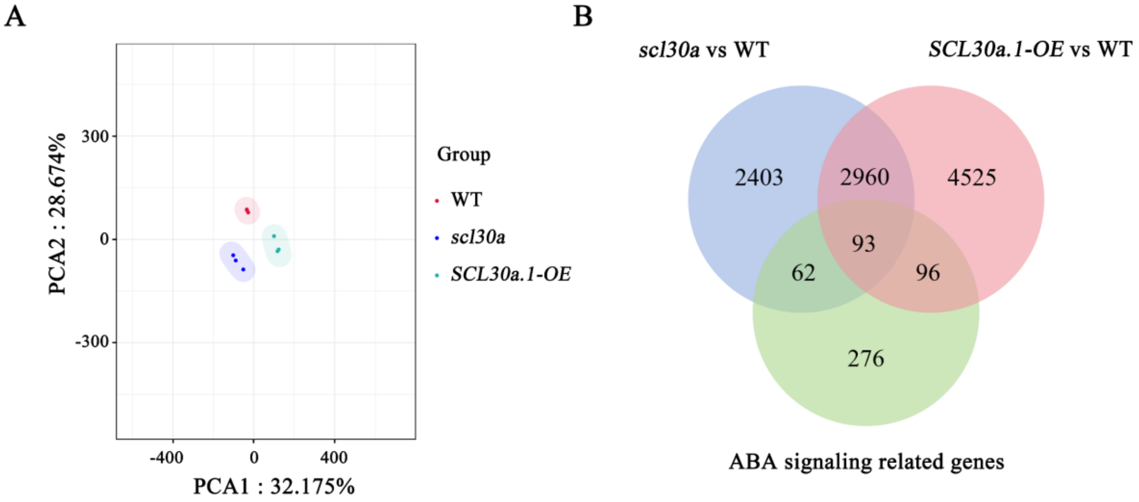


**Figure S4.** Principal component analysis and Venn diagram of differentially expressed genes from transcriptome sequencing

**(A)** Principal component analysis (PCA). PCA of transcriptome samples from WT, *scl30a*, and *SCL30a.1*-OE under ABA treatment. The clustering distribution of each sample is indicated.

**(B)** Venn diagram of differentially expressed genes (DEGs). Overlap of three gene sets: DEGs from the *scl30a* mutant, DEGs from the *SCL30a.1*-OE line, and known ABA signaling pathway-related genes (curated from the literature). The numbers of unique and shared genes in each set are indicated.

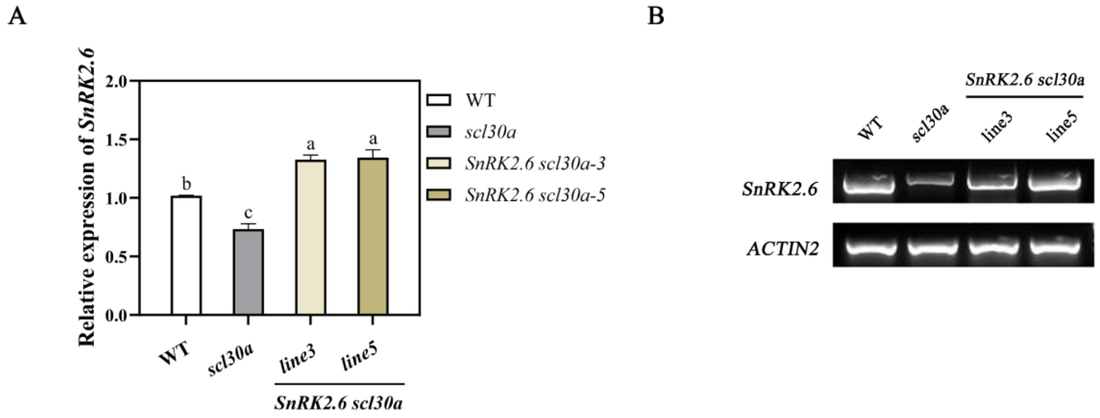


**Figure S5.** Molecular identification of *SnRK2.6* transgenic plants

**(A)** qRT-PCR expression analysis. Expression levels of *SnRK2.6* in WT, *scl30a*, and *SnRK2.6* complementation lines (*SnRK2.6 scl30a*). Data are shown as mean ± SE from at least three independent biological replicates. *ACTIN2* and *GAPDH* were used as reference genes. Different letters indicate significant differences ($p < 0.05$) among genotypes.

**(B)** RT-PCR identification. RT-PCR analysis of *SnRK2.6* transcripts in WT, *scl30a*, and *SnRK2.6/scl30a* complementation lines. *ACTIN2* served as a loading control.
